# Supplementary material for: Hyperbaric oxygen enhances tumor penetration and accumulation of engineered bacteria for synergistic photothermal immunotherapy
Source: Nat Commun. 2024 Jun 17;15:5147. doi: 10.1038/s41467-024-49156-6 (PMC11183253; doi:10.1038/s41467-024-49156-6)
Supplement: Supplementary file 1 — Supplementary Information [file 41467_2024_49156_MOESM1_ESM.pdf]

Hyperbaric oxygen enhances tumor penetration and accumulation of engineered bacteria for synergistic photothermal immunotherapy

Ke-Fei Xu[1], Shun-Yu Wu[1], Zihao Wang[1], Yuxin Guo[1], Ya-Xuan Zhu[2], Chengcheng Li[3], Bai-Hui Shan[1], Xinping Zhang[1], Xiaoyang Liu[1], and Fu-Gen Wu[1]*

[1]*State Key Laboratory of Digital Medical Engineering, Jiangsu Key Laboratory for Biomaterials and Devices, School of Biological Science and Medical Engineering, Southeast University, 2 Southeast University Road, Nanjing 211189, P. R. China*

[2]*Shanghai Tenth People's Hospital, Tongji University School of Medicine, Shanghai 200072, P. R. China.*

[3]*International Innovation Center for Forest Chemicals and Materials and Jiangsu Co-Innovation Center for Efficient Processing and Utilization of Forest Resources, Nanjing Forestry University, Nanjing 210037, P. R. China*

Corresponding author

*Fu-Gen Wu, E-mail: wufg@seu.edu.cn.

Supplementary figures

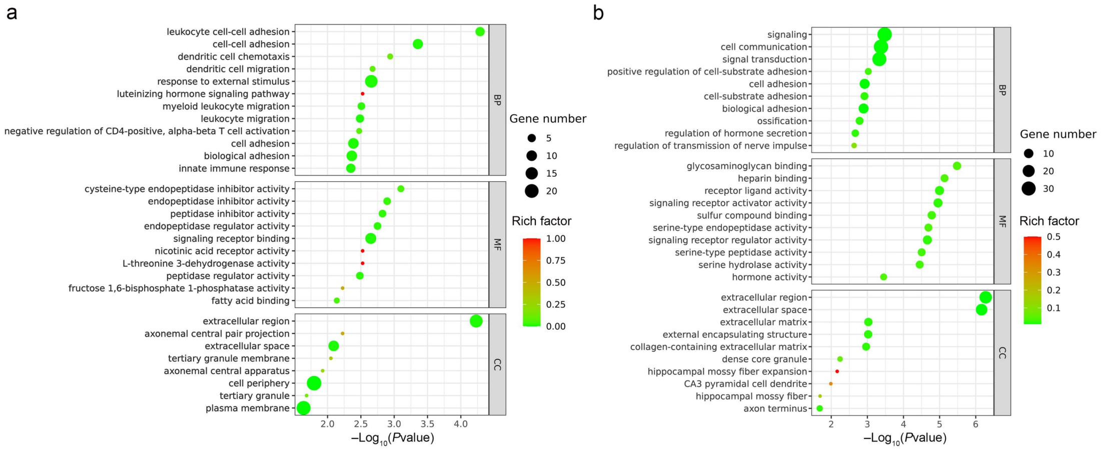

Supplementary Fig. 1 | Dot plot showing the GO enrichment analysis results of top 30 (**a**) upregulated and (**b**) downregulated DEGs in the 4T1 tumors from the mice in the "HBO+" and "HBO–" groups. *n* = 3 mice. Statistical significance was calculated via two-tailed Student's *t*-test.

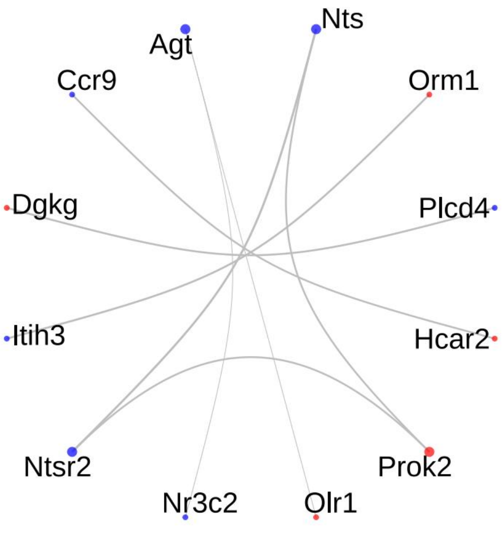

Supplementary Fig. 2 | Protein-protein interaction (PPI) network of the DEGs in the 4T1 tumors from the mice in the "HBO+" and "HBO–" groups.

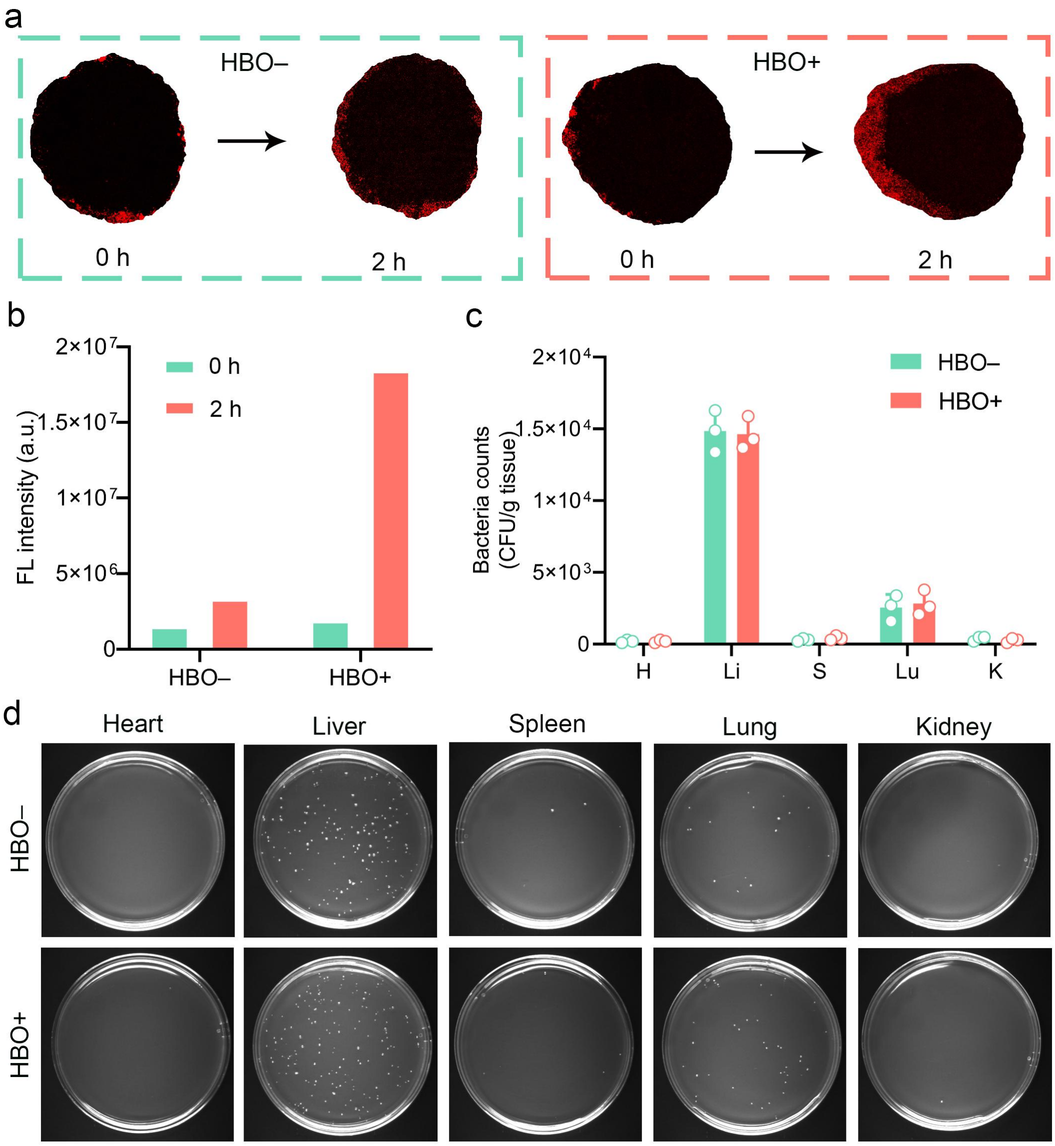

Supplementary Fig. 3 | **a**, Confocal fluorescence images showing the EcN-mCherry-treated MCSs in the absence or presence of HBO treatment (0 or 2 h). **b**, Corresponding quantitative fluorescence intensities of EcN-mCherry inside the 3D tumor spheroids in (**a**). **c**, Quantitative bacterial counts in the major organs from the mice after injection of EcN without/with HBO treatment. Data are presented as mean ± standard deviation (SD). *n* = 3 mice. **d**, Representative photographs of LB plates inoculated with the EcN from the major organs of the mice after bacterial injection. The dilution factor of the original bacterial suspension was 100. Source data are provided as a Source Data file.

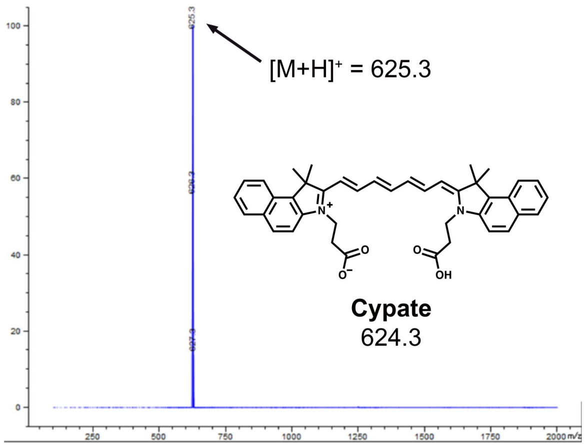

Supplementary Fig. 4 | ESI-MS curve of cypate. Source data are provided as a Source Data file.

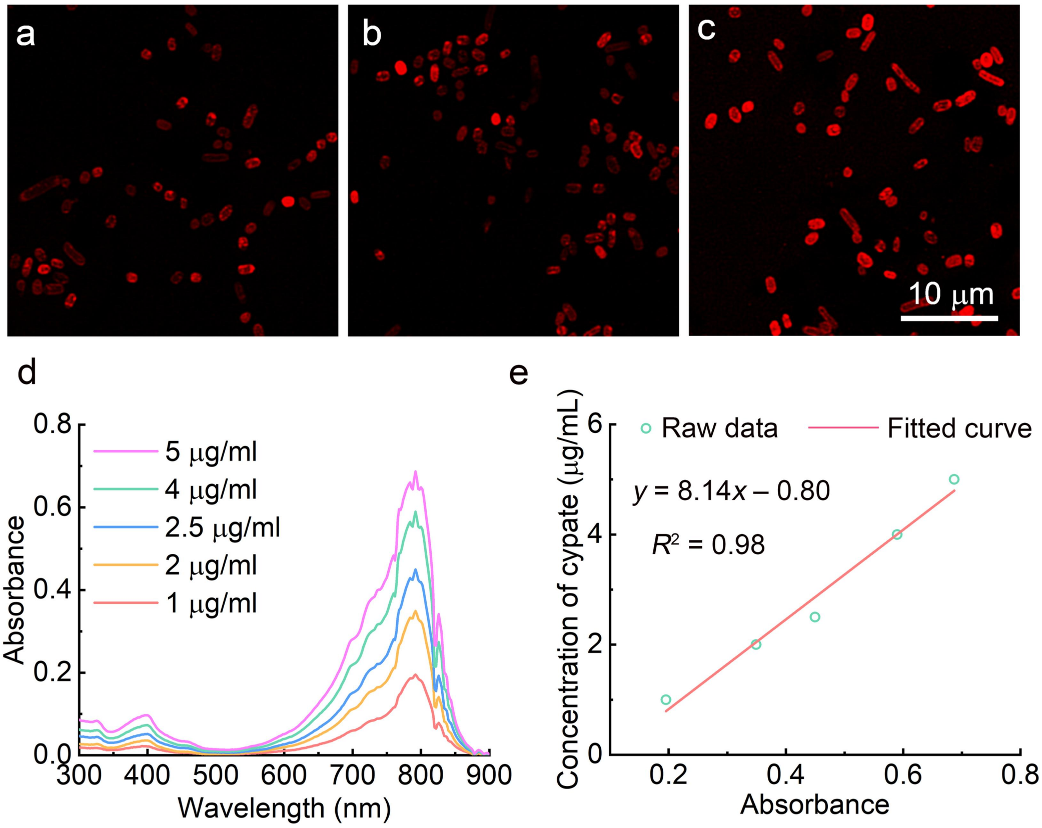

Supplementary Fig. 5 | Confocal fluorescence images of EcN after reacting with (**a**) 500, (**b**) 750, and (**c**) 1000 μg/mL cypate, which was pretreated with EDC•HCl and NHS, respectively. **d**, UV–vis absorption spectra of different concentrations of cypate solution. **e**, Plot of absorption peak at 790 nm versus the concentration of free cypate and the linear calibration. Source data are provided as a Source Data file.

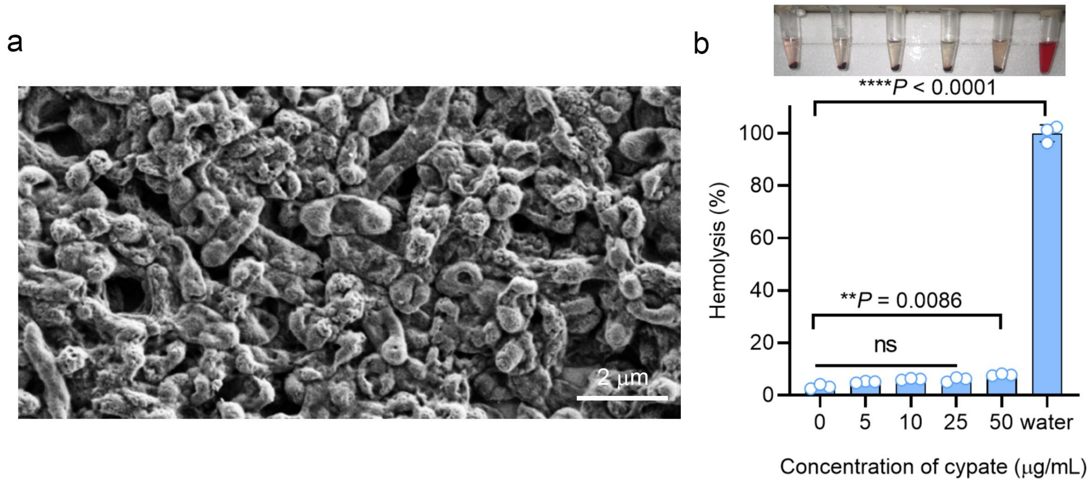

Supplementary Fig. 6 | **a**, SEM image of EcN-cypate after laser irradiation (808 nm, 1 W/cm^2, 10 min). **b**, Hemolysis results of EcN-cypate at various concentrations of cyapte as indicated. RBCs in water were set as the positive control, respectively. Data are presented as mean ± SD. *n* = 3 experimental replicates. Statistical significance in **b** was calculated via one-way analysis of variance (ANOVA) with a Tukey's post-hoc test. ***P* < 0.01, *****P* < 0.0001. "ns" stands for nonsignificant difference. Source data are provided as a Source Data file.

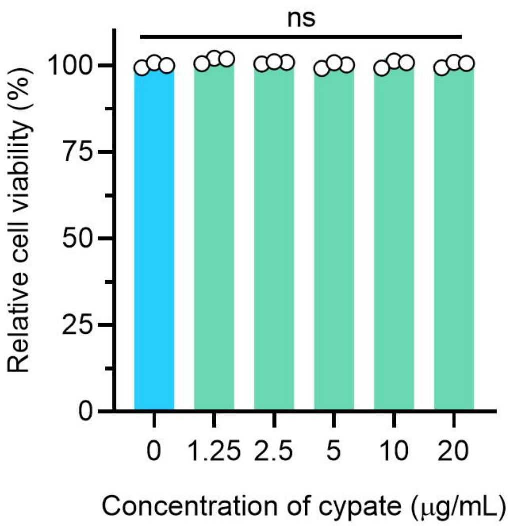

Supplementary Fig. 7 | Relative viabilities of 4T1 cells incubated with different concentrations of EcN-cypate (cypate: 0, 1.25, 2.5, 5, 10, and 20 μg/mL) for 24 h. Data are presented as mean ± SD. *n* = 3 experimental repeats. Statistical significance

was calculated via one-way ANOVA with a Tukey's post-hoc test. "ns" stands for nonsignificant difference. Source data are provided as a Source Data file.

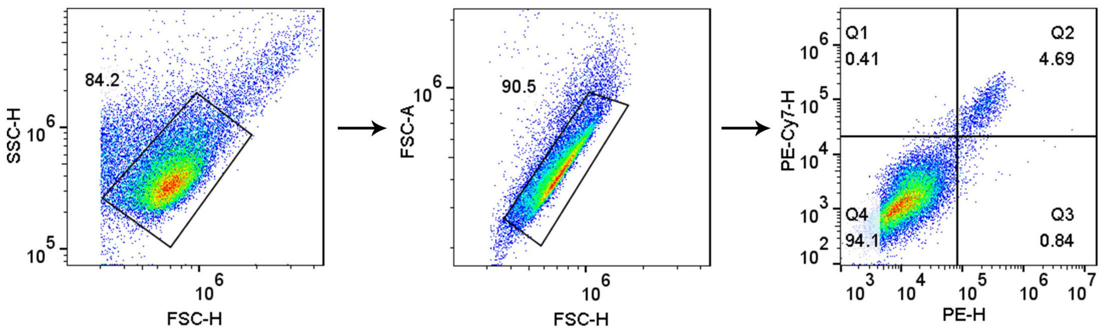

Supplementary Fig. 8 | Representative flow cytometry gating strategy for mature DCs (CD11c^{+}CD80^{+}CD86^{+}).

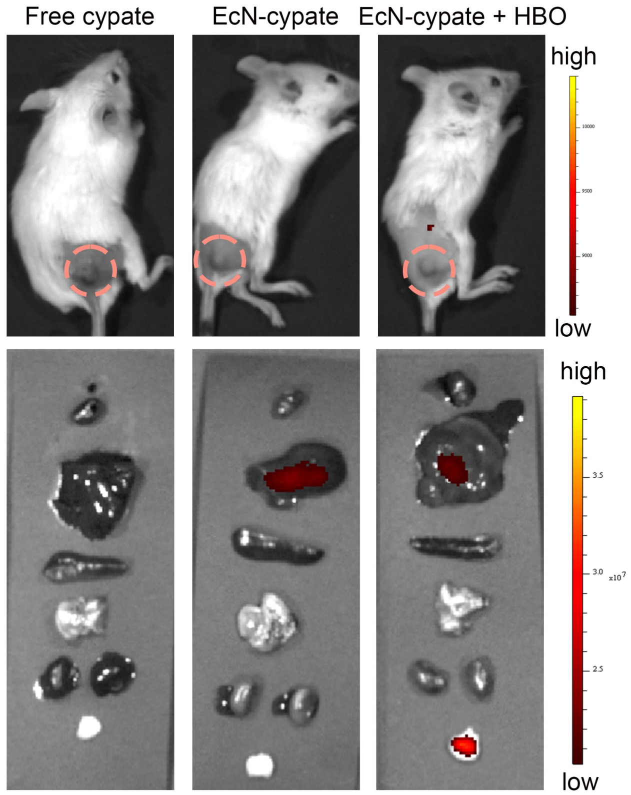

Supplementary Fig. 9 | In vivo and ex vivo distributions of cypate at day 7 post intravenous injection of cypate or EcN-cypate (cypate dose: 10 mg/kg). "EcN-cypate + HBO" group: The mice were treated with HBO (1.5 ATA, 2 h) at 12, 36, and 60 h post injection.

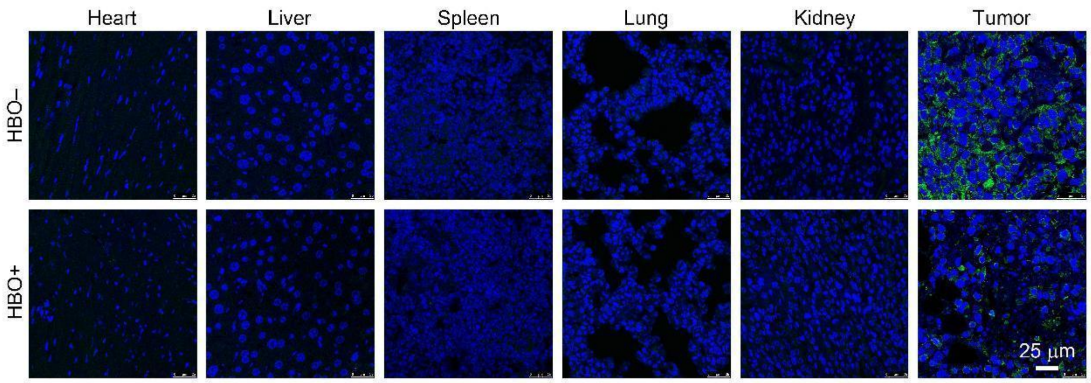

Supplementary Fig. 10 | Representative immunofluorescence images showing the HIF-1α expression in major organs and tumors derived from the tumor-bearing mice with/without HBO treatment. This experiment was repeated for three times independently with similar results.

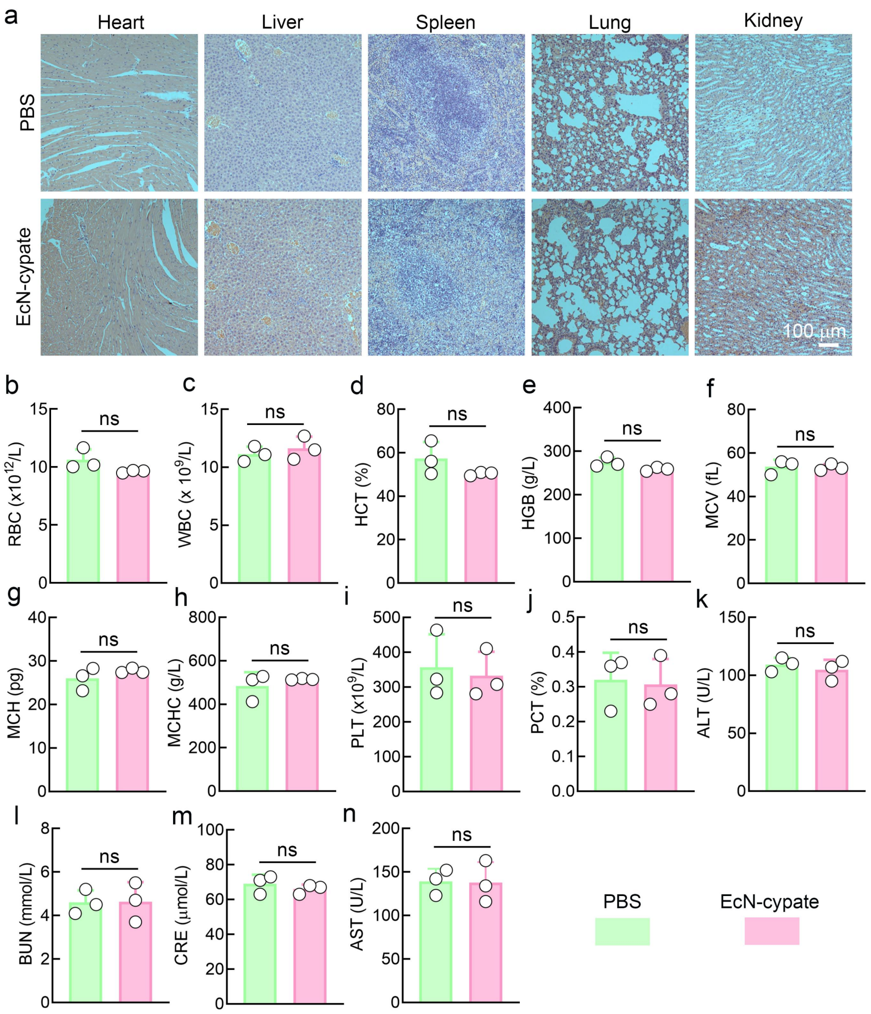

Supplementary Fig. 11 | **Biosafety evaluation of EcN-cypate. a**, Representative

H&E-stained tissue slices of major organs in healthy BALB/c mice (without tumor inoculation) that were sacrificed at 14 d after intravenous injection of PBS or EcN-cypate suspension (cypate dose: 10 mg/kg). **b–n**, Hemanalysis and biochemical analysis results of the healthy BALB/c mice sacrificed at day 14 after injection of PBS or EcN-cypate suspension (cypate dose: 10 mg/kg). Data are presented as mean ± SD. n = 3 mice. Statistical data in **b–n** are calculated via two-tailed Student's t-test. "ns" stands for nonsignificant difference. Source data are provided as a Source Data file.

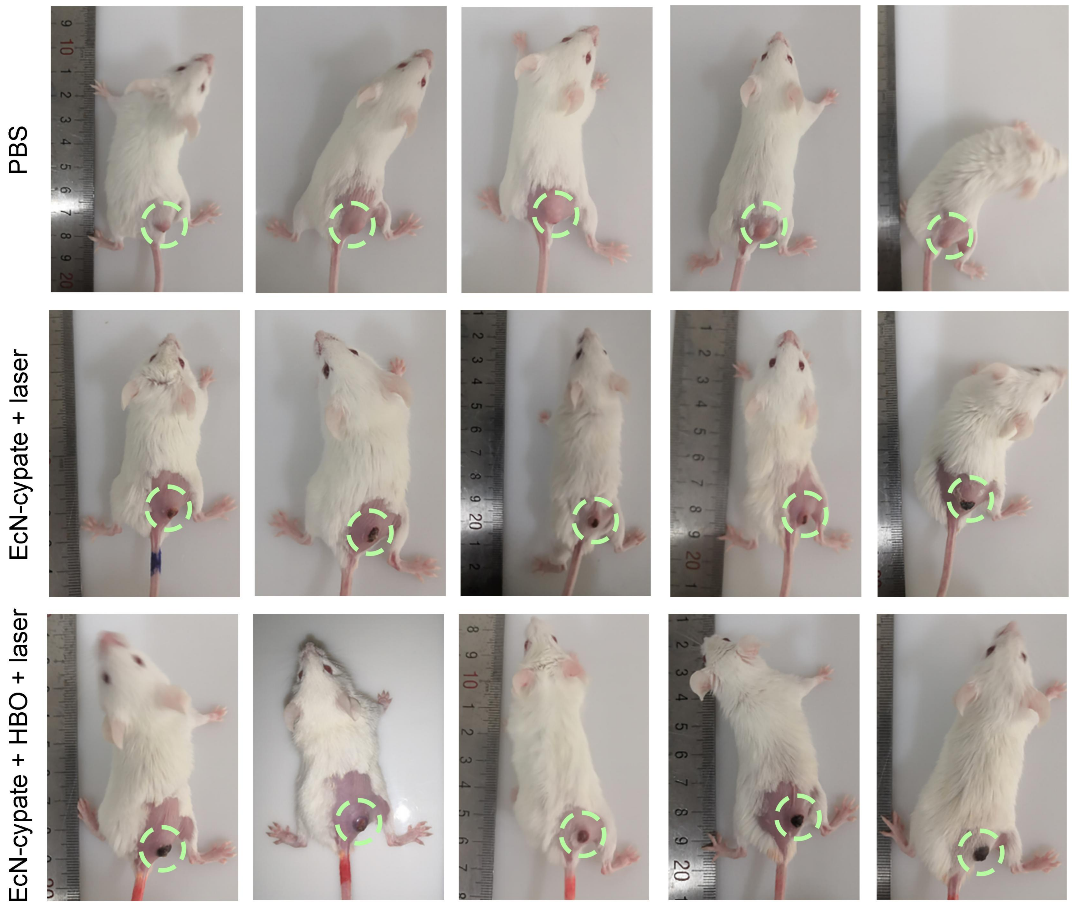

Supplementary Fig. 12 | Photographs of the 4T1 tumor-bearing BALB/c mice subjected to different treatments at 48 h post injection. The tumor regions were marked by circles.

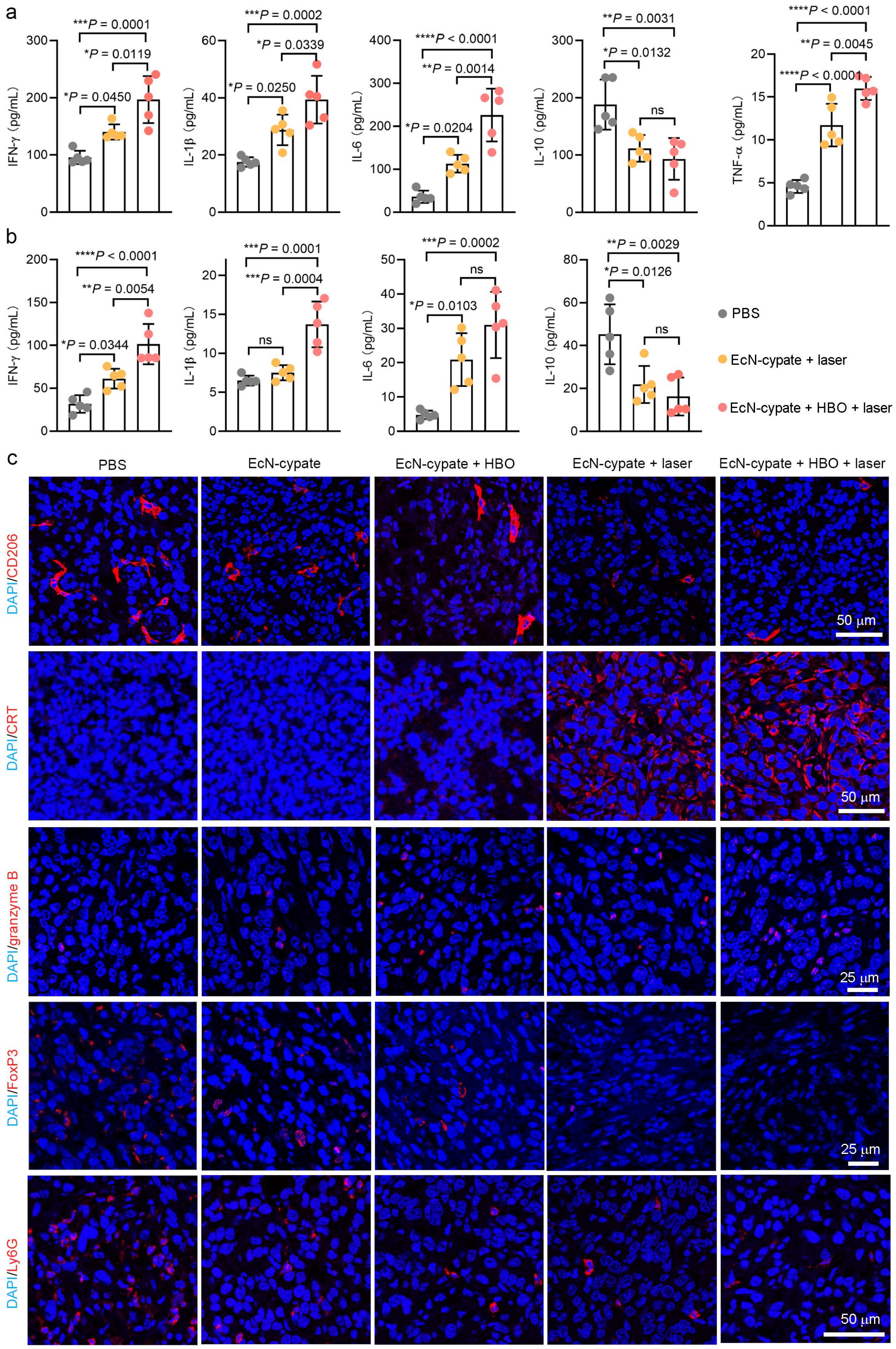

Supplementary Fig. 13 | Analysis of the intratumoral immunostimulatory effect triggered by HBO-enhanced PTT in the 4T1 tumor model. a, Intratumoral levels of IFN-γ, IL-1β, IL-6, IL-10, and TNF-α analyzed by ELISA. Data are presented as

mean ± SD. $n = 5$ mice. **b**, Serum levels of IFN-γ, IL-1β, IL-6, and IL-10 analyzed by ELISA. Data are presented as mean ± SD. $n = 5$ mice. Statistical significance in **a** and **b** was calculated via one-way ANOVA with a Tukey's post-hoc test. $^{*}P < 0.05$, $^{**}P < 0.01$, $^{***}P < 0.001$, $^{****}P < 0.0001$. "ns" stands for nonsignificant difference. **c**, Representative immunofluorescence staining results of the CD206, CRT, granzyme B, FoxP3, and Ly6G in the tumor tissue slices from the 4T1 tumor-bearing BALB/c mice after different treatments as indicated. This experiment was repeated for three times independently with similar results. Source data are provided as a Source Data file.

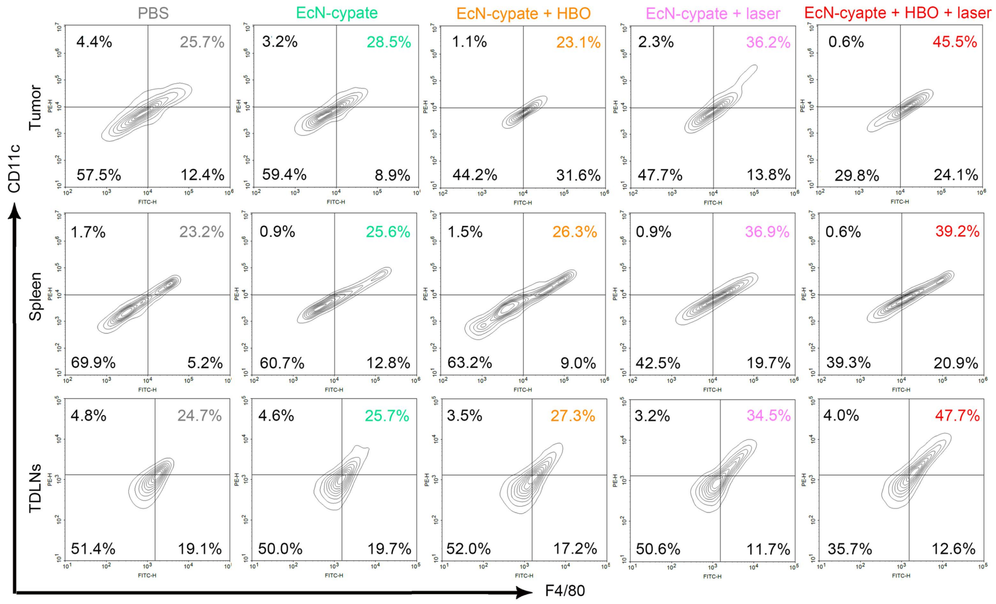

Supplementary Fig. 14 | Representative flow cytometric plots of the M1-like macrophages (F4/80^{+}CD11c^{+}) in the tumors, spleens, and TDLNs retrieved from the 4T1-bearing BALB/c mice at day 8 post injection.

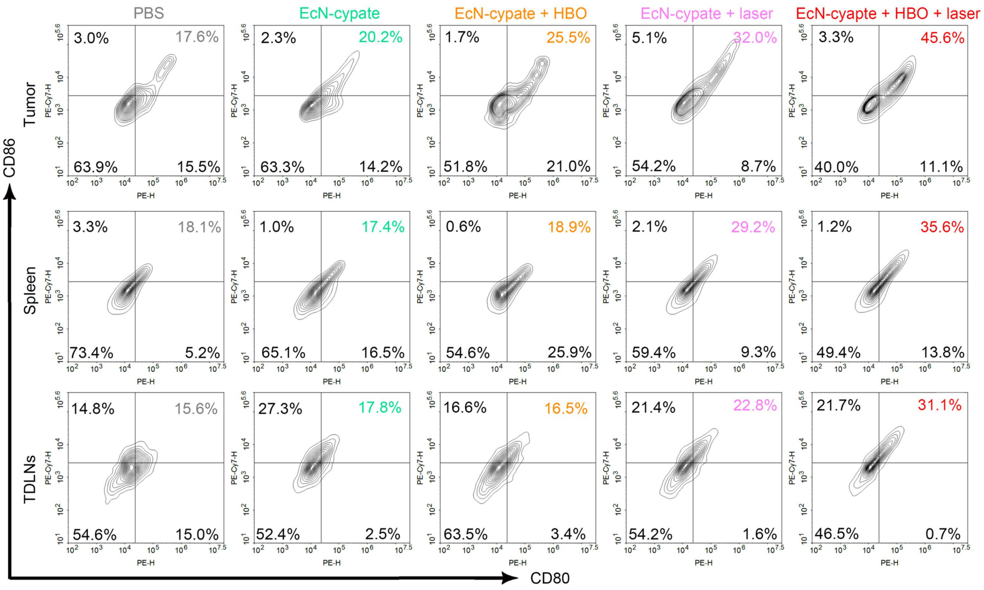

Supplementary Fig. 15 | Representative flow cytometric plots of mature DCs ($CD11c^{+}CD80^{+}CD86^{+}$) in the tumors, spleens, and TDLNs retrieved from the 4T1-bearing BALB/c mice at day 8 post injection.

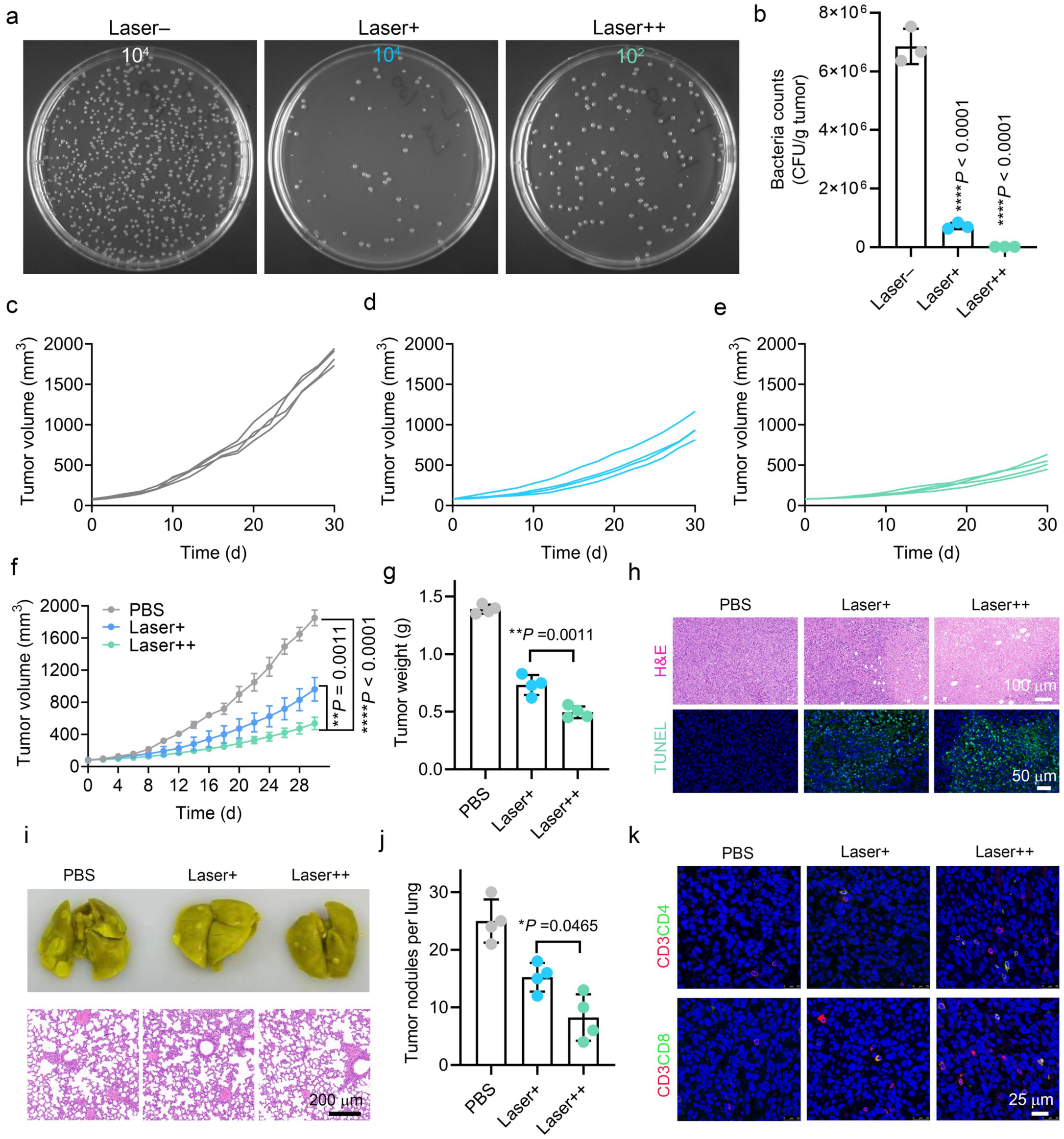

Supplementary Fig. 16 | **a**, Representative photographs showing EcN colonization behaviors inside the tumors from the 4T1 tumor-bearing mice with different treatments. The numbers (10^4, 10^2) in the images indicated the dilution factors of the original bacterial suspensions. **b**, Corresponding quantitative bacteria counts in (**a**). Data are presented as mean ± SD. *n* = 3 mice. Detailed tumor growth curves of each 4T1 tumor-bearing mouse in the (**c**) PBS, (**d**) "Laser+", and (**e**) "Laser++" groups. **f**, Tumor volume changes of tumors in different groups. **g**, Tumor weights in different groups at day 30. Data are presented as mean ± SD. *n* = 4 mice. **h**, H&E- and TUNEL assay kit-stained tumor slices of 4T1 tumor-bearing mice after different treatments. **i**, Respective photographs and H&E staining images of the lungs collected from

different groups. **j**, Quantification results of the tumor nodules in the lungs collected from different groups. Data are presented as mean ± SD. *n* = 4 mice. **k**, Representative confocal fluorescence images of the immunofluorescence staining results of $CD3^+CD4^+$ and $CD3^+CD8^+$ T cells in tumor slices. This experiment was repeated for three times independently with similar results. "Laser–": The mice were i.v. injected with EcN-cypate for 24 h. "Laser+": The mice were irradiated by laser (808 nm, 1 W/cm^2, 15 min) at 24 h post injection of EcN-cypate. "Laser++": The mice were irradiated by laser (808 nm, 1 W/cm^2, 15 min) at 24 and 48 h post injection of EcN-cypate. Statistical data in (**b**), (**f**), (**g**), and (**j**) are analyzed by one-way ANOVA (*$P < 0.05$, **$P < 0.01$, ****$P < 0.0001$). Source data are provided as a Source Data file.

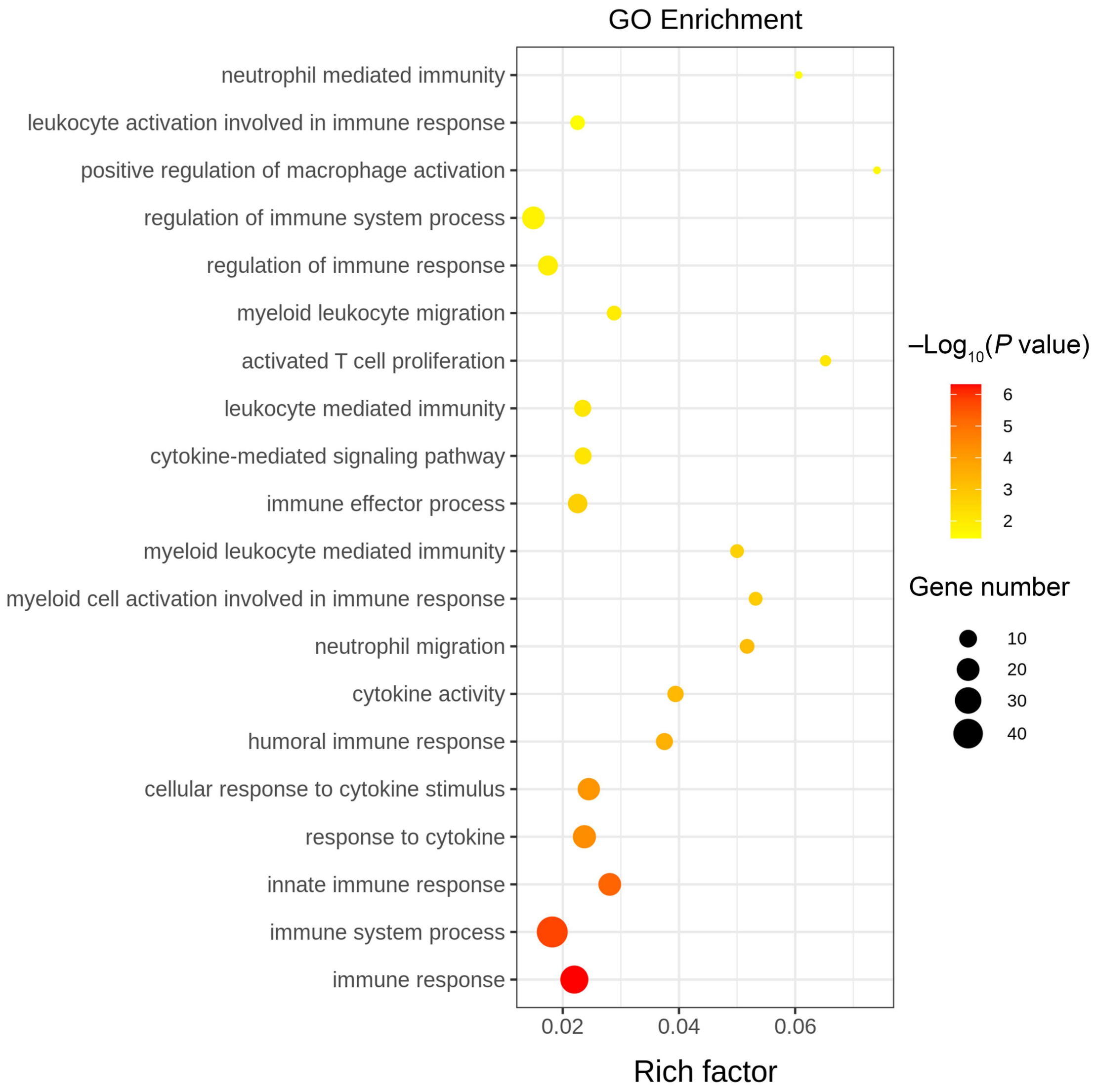

Supplementary Fig. 17 | Dot plot showing the GO enrichment analysis results of some selected DEGs in the 4T1 tumors from the mice after PBS (control) or "EcN-cypate + HBO + laser" treatment. n = 3 mice. Statistical significance was calculated via two-tailed Student's t-test.

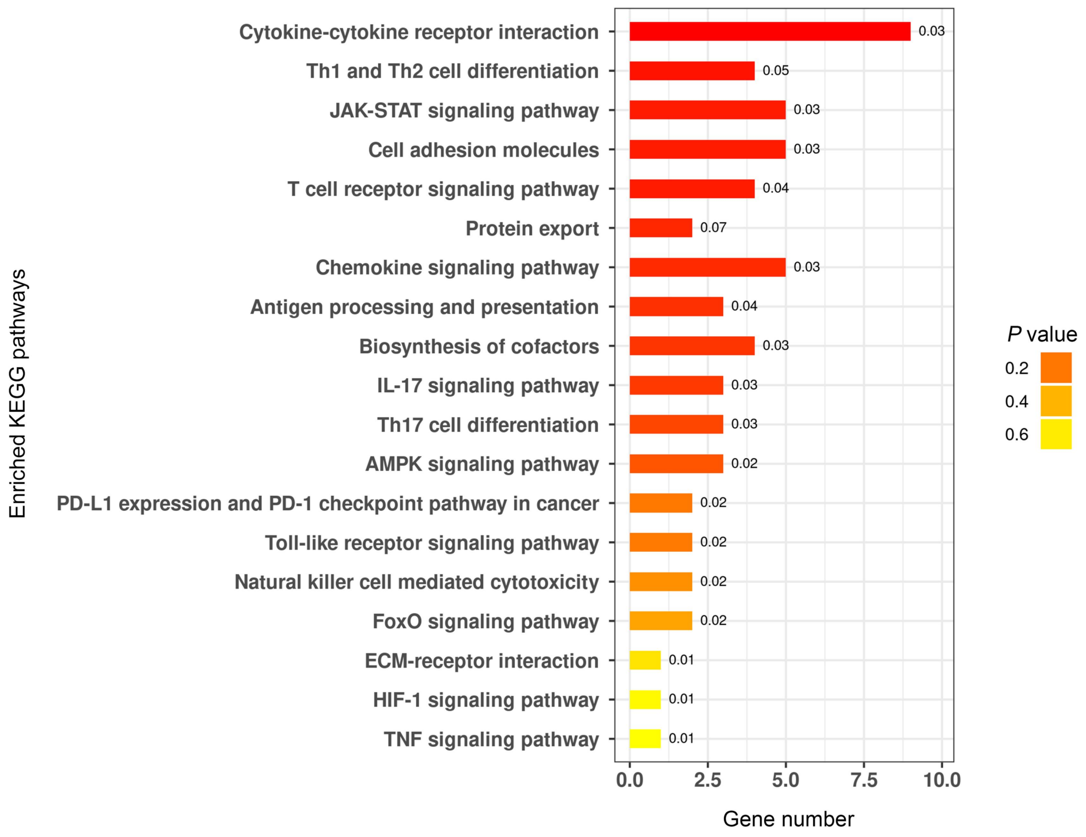

Supplementary Fig. 18 | Histogram showing the KEGG enrichment analysis results of some selected DEGs in the 4T1 tumors from the mice after PBS (control) or "EcN-cypate + HBO + laser" treatment. n = 3 mice. Statistical significance was calculated via two-tailed Student's t-test.

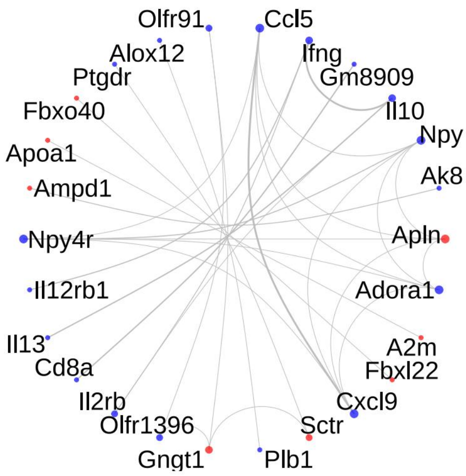

Supplementary Fig. 19 | PPI network of the DEGs in the 4T1 tumors from the mice in the control and "EcN-cypate + HBO + laser" groups.

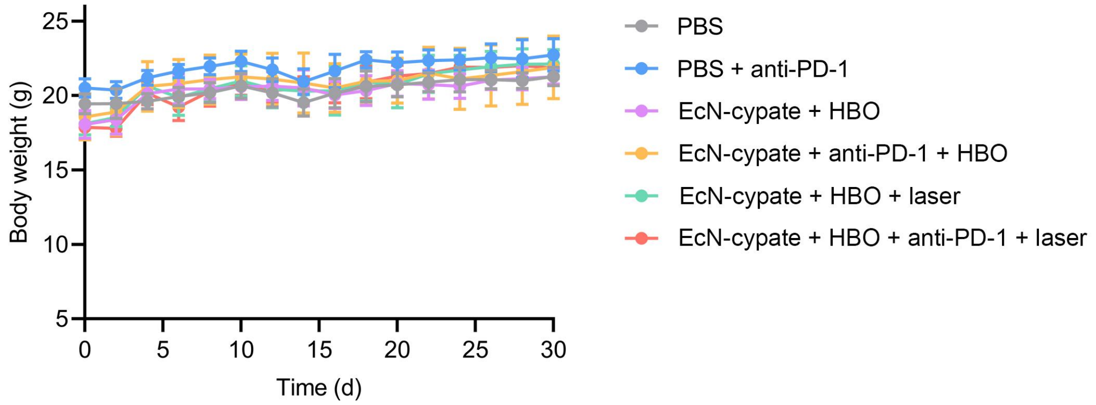

Supplementary Fig. 20 | Body weight fluctuations of the mice in different groups. Data are presented as mean ± SD. *n* = 5 mice. Source data are provided as a Source Data file.

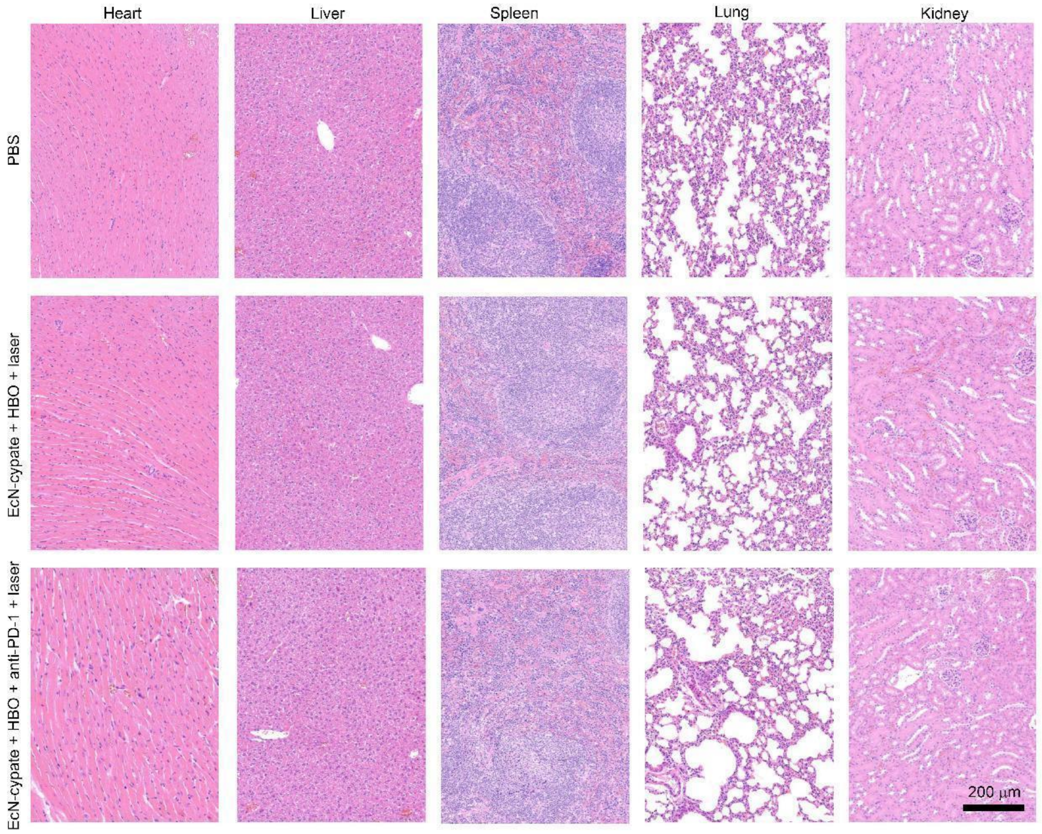

Supplementary Fig. 21 | Representative H&E-stained tissue slices of major organs in the 4T1 tumor-bearing BALB/c mice that were sacrificed at 2 d after intravenous injection of PBS or EcN-cypate suspension (cypate dose: 10 mg/kg). The mice in the "EcN-cypate + HBO + laser" and "EcN-cypate + HBO + anti-PD-1 + laser" groups were irradiated by an 808 nm laser (1 W/cm^2, 15 min) at 24 and 48 h post injection of EcN-cypate.

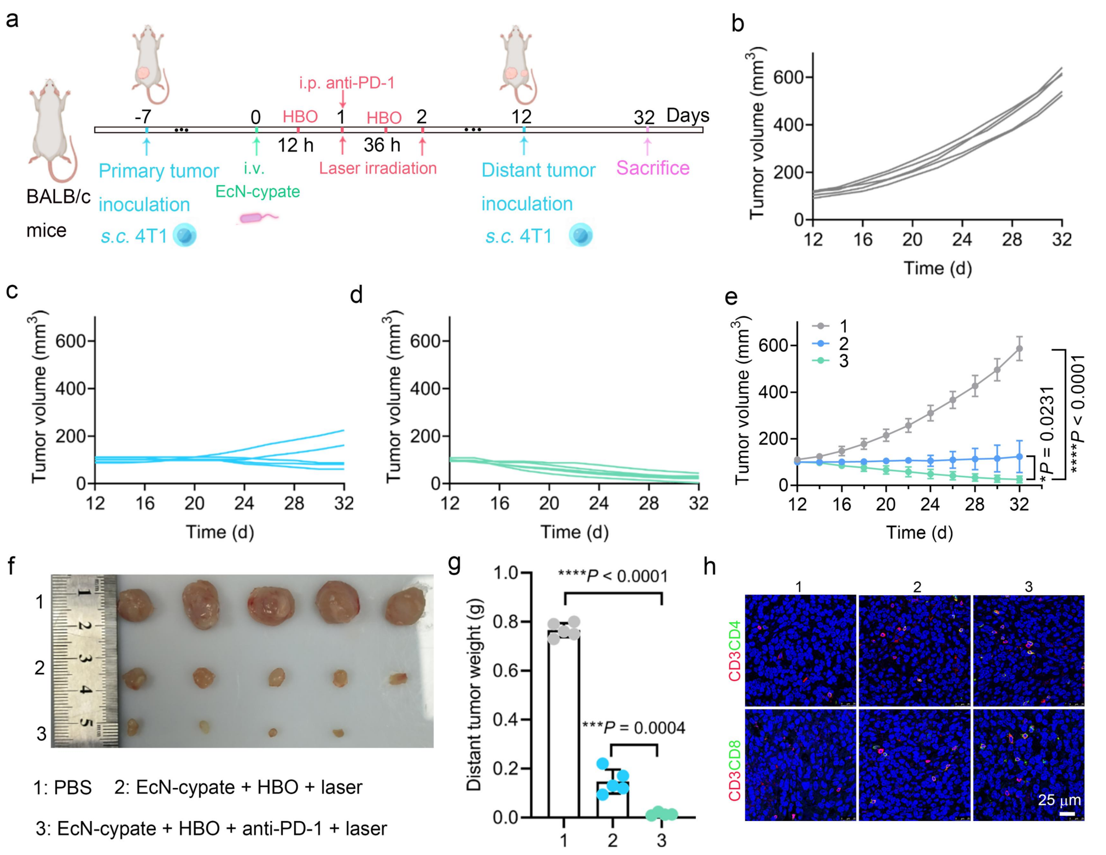

Supplementary Fig. 22 | **a**, Schematic illustration of the animal experimental design for evaluating the therapeutic and immunostimulation outcomes of different groups in rechallenge tumor models. Detailed growth curves of each distant tumors in the (**b**) PBS, (**c**) "EcN-cypate + HBO + laser", and (**d**) "EcN-cypate + HBO + anti-PD-1 + laser" groups, respectively. **e**, Average tumor volume changes of distant tumors in different groups. Data are presented as mean ± SD. n = 5 mice. **f**, Photographs showing the distant tumor tissues collected from the 4T1 tumor-bearing mice at day 32 after different treatments. **g**, Distant tumor weights in different groups at day 32. Data are presented as mean ± SD. n = 5 mice. **h**, Representative confocal fluorescence images of the immunofluorescence staining results of $CD3^+CD4^+$ and $CD3^+CD8^+$ T cells in distant tumor slices. This experiment was repeated for three times independently with similar results. Statistical data are analyzed by one-way ANOVA (*P < 0.05, ***P < 0.001, ****P < 0.0001). Source data are provided as a Source Data file.
