# Supplementary material for: Hyperbaric oxygen enhances tumor penetration and accumulation of engineered bacteria for synergistic photothermal immunotherapy
Source: Nat Commun. 2024 Jun 17;15:5147. doi: 10.1038/s41467-024-49156-6 (PMC11183253; doi:10.1038/s41467-024-49156-6)
Supplement: Supplementary file 3 — Reporting Summary [file 41467_2024_49156_MOESM3_ESM.pdf]

nature portfolio

Corresponding author(s): Fu-Gen Wu

Last updated by author(s): May 1, 2024

Reporting Summary

Nature Portfolio wishes to improve the reproducibility of the work that we publish. This form provides structure for consistency and transparency in reporting. For further information on Nature Portfolio policies, see our Editorial Policies and the Editorial Policy Checklist.

Statistics

For all statistical analyses, confirm that the following items are present in the figure legend, table legend, main text, or Methods section.

n/a	Confirmed	
☐	☒	The exact sample size (*n*) for each experimental group/condition, given as a discrete number and unit of measurement
☐	☒	A statement on whether measurements were taken from distinct samples or whether the same sample was measured repeatedly
☐	☒	The statistical test(s) used AND whether they are one- or two-sided<br>*Only common tests should be described solely by name; describe more complex techniques in the Methods section.*
☐	☒	A description of all covariates tested
☐	☒	A description of any assumptions or corrections, such as tests of normality and adjustment for multiple comparisons
☐	☒	A full description of the statistical parameters including central tendency (e.g. means) or other basic estimates (e.g. regression coefficient) AND variation (e.g. standard deviation) or associated estimates of uncertainty (e.g. confidence intervals)
☐	☒	For null hypothesis testing, the test statistic (e.g. *F*, *t*, *r*) with confidence intervals, effect sizes, degrees of freedom and *P* value noted<br>*Give P values as exact values whenever suitable.*
☒	☐	For Bayesian analysis, information on the choice of priors and Markov chain Monte Carlo settings
☒	☐	For hierarchical and complex designs, identification of the appropriate level for tests and full reporting of outcomes
☒	☐	Estimates of effect sizes (e.g. Cohen's *d*, Pearson's *r*), indicating how they were calculated

Our web collection on statistics for biologists contains articles on many of the points above.

Software and code

Policy information about availability of computer code

Data collection	No software was used.
Data analysis	All statistical analyses were performed using GraphPad Prism 9, Origin 2018, or Excel 2019. NovoExpress (version 1.5) and FlowJo (version 10) were used for flow cytometric analysis. The fluorescence signals of the cells in the images were analyzed by ImageJ (version 1.52a). The sequencing data of the transcriptomic analyses were analyzed using the FastQ Screen Trimmomatic (version 0.23.2) and HISAT2 (version 2.2.1).

For manuscripts utilizing custom algorithms or software that are central to the research but not yet described in published literature, software must be made available to editors and reviewers. We strongly encourage code deposition in a community repository (e.g. GitHub). See the Nature Portfolio guidelines for submitting code & software for further information.

Data

Policy information about availability of data

All manuscripts must include a data availability statement. This statement should provide the following information, where applicable:

- Accession codes, unique identifiers, or web links for publicly available datasets
- A description of any restrictions on data availability
- For clinical datasets or third party data, please ensure that the statement adheres to our policy

The main data supporting the findings in this study are available within the paper and its Supplementary Information. All data generated during the study are

available from the corresponding author on reasonable request. Source data are provided with this paper. The sequencing data of the transcriptomic analyses in this study are available from the Sequence Read Archive (SRA) Run Selector of the National Center Biotechnology Information (NCBI) database with the NCBI BioProject accession number PRJNA1105037 (https://www.ncbi.nlm.nih.gov/Traces/study/?acc=PRJNA1105037&o=acc_s%3Aa). The sequencing data of the transcriptomic analyses were analyzed using the FastQ Screen Trimmomatic (version 0.23.2) and HISAT2 (version 2.2.1). GO (http://www.geneontology.org/) and KEGG (http://www.genome.jp/kegg/pathway.html) analyses were explored to evaluate the biological function of the DEGs. PPI network of DEGs was analyzed via the STRING database (http://string-db.org/).

Research involving human participants, their data, or biological material

Policy information about studies with human participants or human data. See also policy information about sex, gender (identity/presentation), and sexual orientation and race, ethnicity and racism.

Reporting on sex and gender	*Use the terms sex (biological attribute) and gender (shaped by social and cultural circumstances) carefully in order to avoid confusing both terms. Indicate if findings apply to only one sex or gender; describe whether sex and gender were considered in study design; whether sex and/or gender was determined based on self-reporting or assigned and methods used. Provide in the source data disaggregated sex and gender data, where this information has been collected, and if consent has been obtained for sharing of individual-level data; provide overall numbers in this Reporting Summary. Please state if this information has not been collected. Report sex- and gender-based analyses where performed, justify reasons for lack of sex- and gender-based analysis.*
Reporting on race, ethnicity, or other socially relevant groupings	*Please specify the socially constructed or socially relevant categorization variable(s) used in your manuscript and explain why they were used. Please note that such variables should not be used as proxies for other socially constructed/relevant variables (for example, race or ethnicity should not be used as a proxy for socioeconomic status). Provide clear definitions of the relevant terms used, how they were provided (by the participants/respondents, the researchers, or third parties), and the method(s) used to classify people into the different categories (e.g. self-report, census or administrative data, social media data, etc.) Please provide details about how you controlled for confounding variables in your analyses.*
Population characteristics	*Describe the covariate-relevant population characteristics of the human research participants (e.g. age, genotypic information, past and current diagnosis and treatment categories). If you filled out the behavioural & social sciences study design questions and have nothing to add here, write "See above."*
Recruitment	*Describe how participants were recruited. Outline any potential self-selection bias or other biases that may be present and how these are likely to impact results.*
Ethics oversight	*Identify the organization(s) that approved the study protocol.*

Note that full information on the approval of the study protocol must also be provided in the manuscript.

Field-specific reporting

Please select the one below that is the best fit for your research. If you are not sure, read the appropriate sections before making your selection.

☒ Life sciences ☐ Behavioural & social sciences ☐ Ecological, evolutionary & environmental sciences

For a reference copy of the document with all sections, see nature.com/documents/nr-reporting-summary-flat.pdf

Life sciences study design

All studies must disclose on these points even when the disclosure is negative.

Sample size	No sample size calculation was performed. Sample sizes were based on the authors' experience with in vitro and in vivo studies, as published in many studies. At least 3 technical and/or biological replicates for each sample were conducted. The sample sizes were indicated in the legend of each figure and determined as the minimal values to lower the cost and be sufficient to obtain statistically significant difference between experimental groups (n = 3-5).
Data exclusions	No data were excluded.
Replication	We repeated the experimental results 3 times to confirm the reproducibility. Details of experimental replicates were given in the figure legends.
Randomization	Animal groups were randomized by body weight. In the other experiments, samples were randomly assigned to experimental groups.
Blinding	All the investigators were blinded to group allocation during data collection and analysis.

Reporting for specific materials, systems and methods

We require information from authors about some types of materials, experimental systems and methods used in many studies. Here, indicate whether each material, system or method listed is relevant to your study. If you are not sure if a list item applies to your research, read the appropriate section before selecting a response.

Materials & experimental systems

n/a	Involved in the study
☐	☒ Antibodies
☐	☒ Eukaryotic cell lines
☒	☐ Palaeontology and archaeology
☐	☒ Animals and other organisms
☒	☐ Clinical data
☒	☐ Dual use research of concern
☒	☐ Plants

Methods

n/a	Involved in the study
☒	☐ ChIP-seq
☐	☒ Flow cytometry
☒	☐ MRI-based neuroimaging

Antibodies

Antibodies used

All the antibodies were diluted 200 times and used following the suppliers' protocols.

Rabbit anti-HIF-1α polyclonal antibody (cat. no. bs-20399R) and FITC-labeled goat anti-mouse IgG antibody (cat. no. bs-0296G-FITC) were obtained from Bioss Antibodies (Beijing, China). PD-1 monoclonal antibody (cat. no. BP1046) was obtained from BioXcell (New Hampshire, USA). CRT polyclonal antibody (cat. no. 27298-1-AP), fibronectin polyclonal antibody (cat. no. 15613-1-AP), and HMGB1 polyclonal antibody (cat. no. 10829-1-AP) were purchased from Proteintech (Wuhan, China). Anti-mouse CD4 (cat. no.GB15064), anti-mouse CD3 (cat. no. GB12014), anti-mouse CD8 (cat. no. GB15068), anti-mouse granzyme B (cat. no. GB12093), anti-mouse Ly6G (cat. no. GB11229), anti-mouse FoxP3 (cat. no. GB11093), anti-mouse CD206 (cat. no. GB13438), and Cy3-labeled goat anti-rabbit IgG antibody (cat. no. GB21303) were obtained from Wuhan Servicebio Technology Co., Ltd. Anti-mouse CD3-PE (cat. no. 12-0032-82), anti-mouse CD4-FITC (cat. no. 11-0041-82), anti-mouse CD8-PE-Cy7 (cat. no. 25-0081-81), anti-mouse CD11c-FITC (cat. no. 11-0114-82), anti-mouse CD80-PE (cat. no. 12-0801-82), and anti-mouse CD86-PE-Cy7 (cat. no. 25-0862-82) were bought from Invitrogen (Carlsbad, USA). Anti-mouse CD11c-PE (cat. no. 117308) and anti-mouse F4/80-FITC (cat. no.124611) were purchased from Biolegend (San Diego, USA).

Validation

All antibodies were verified by the supplier and have been quality tested. All validation statements can be found in the respective antibody website:
1.Rabbit anti-HIF-1α polyclonal antibody: https://biosscn.com.cn/index.php?controller=site&action=products&id=2166
2.FITC-labeled goat anti-mouse IgG antibody: http://www.bioss.com.cn/prolook_03.asp?id=AF08169606011198&pro37=4
3.PD-1 monoclonal antibody: https://bxcell.com/product/m-cd279/
4.CRT polyclonal antibody: https://www.ptgcn.com/products/Calreticulin-Antibody-27298-1-AP.htm
5.Fibronectin polyclonal antibody: https://www.ptgcn.com/products/FN1-Antibody-15613-1-AP.htm
6.HMGB1 polyclonal antibody: https://www.ptgcn.com/products/HMGB1-Antibody-10829-1-AP.htm
7.Anti-mouse CD4: https://www.servicebio.cn/goodsdetail?id=13685
8.Anti-mouse CD3: https://www.servicebio.cn/goodsdetail?id=14187
9.Anti-mouse CD8: https://www.servicebio.cn/goodsdetail?id=13908
10.Anti-mouse granzyme B: https://www.servicebio.cn/goodsdetail?id=14658
11.Anti-mouse Ly6G: https://www.servicebio.cn/goodsdetail?id=1454
12.Anti-mouse FoxP3: https://www.servicebio.cn/goodsdetail?id=1374
13.Anti-mouse CD206: https://www.servicebio.cn/goodsdetail?id=6621
14.Cy3-labeled goat anti-rabbit IgG antibody: https://www.servicebio.cn/goodsdetail?id=253
15.Anti-mouse CD3-PE: https://www.thermofisher.cn/cn/zh/antibody/product/CD3-Antibody-clone-17A2-Monoclonal/12-0032-82
16. Anti-mouse CD4-FITC: https://www.thermofisher.cn/cn/zh/antibody/product/CD4-Antibody-clone-GK1-5-Monoclonal/11-0041-82
17.Anti-mouse CD8-PE-Cy7: https://www.thermofisher.cn/cn/zh/antibody/product/CD8a-Antibody-clone-53-6-7-Monoclonal/25-0081-81
18.Anti-mouse CD11c-FITC: https://www.thermofisher.cn/cn/zh/antibody/product/CD11c-Antibody-clone-N418-Monoclonal/11-0114-82
19.Anti-mouse CD80-PE: https://www.thermofisher.cn/cn/zh/antibody/product/CD80-B7-1-Antibody-clone-16-10A1-Monoclonal/12-0801-82
20.Anti-mouse CD86-PE-Cy7: https://www.thermofisher.cn/cn/zh/antibody/product/CD86-B7-2-Antibody-clone-GL1-Monoclonal/25-0862-82
21.Anti-mouse CD11c-PE: https://www.biolegend.com/en-us/products/pe-anti-mouse-cd11c-antibody-1816
22.Anti-mouse F4/80-FITC: https://www.biolegend.com/en-us/search-results/fitc-anti-mouse-f4-80-antibody-4067

Eukaryotic cell lines

Policy information about cell lines and Sex and Gender in Research

Cell line source(s)

4T1 cells were obtained from KeyGEN BioTECH, China. Escherichia coli Nissle 1917 (EcN) was purchased from BioSci Co., Ltd. (China). Primary bone marrow-derived dendritic cells (BMDCs) were collected from female BALB/c mice and cultured by following a standard protocol.

Authentication

Identity of the cell lines was frequently checked by their morphological features but have not been authenticated by the short tandem repeat (STR) profiling.

Mycoplasma contamination

All cell lines were tested for mycoplasma contamination. No mycoplasma contamination was found.

Commonly misidentified lines (See ICLAC register)

No commonly misidentified cell lines were used in this study.

Animals and other research organisms

Policy information about studies involving animals; ARRIVE guidelines recommended for reporting animal research, and Sex and Gender in Research

Laboratory animals	Female BALB/c mice (6–8 weeks) were purchased from Yangzhou University Medical Center (Yangzhou, China). Mice (n = 5/group) were housed in ventilated cage (humidity: 40–70%) with 12 h dark–light cycles at constant room temperature. All mice had access to food and water ad libitum.
Wild animals	The study did not involve wild animals.
Reporting on sex	We did not select mice for each experiment on the basis of sex.
Field-collected samples	The study did not involve samples collected from the field.
Ethics oversight	All the animal experiments were carried out in accordance with the permission from the ethics committee of Southeast University (Nanjing, China) with an approval number of 20221010001. All the animal experiments were conducted in compliance with the Regulations for the Administration of Affairs Concerning Experimental Animals of China. All the animal experiments complied with institutional guidelines.

Note that full information on the approval of the study protocol must also be provided in the manuscript.

Plants

Seed stocks	*Report on the source of all seed stocks or other plant material used. If applicable, state the seed stock centre and catalogue number. If plant specimens were collected from the field, describe the collection location, date and sampling procedures.*
Novel plant genotypes	*Describe the methods by which all novel plant genotypes were produced. This includes those generated by transgenic approaches, gene editing, chemical/radiation-based mutagenesis and hybridization. For transgenic lines, describe the transformation method, the number of independent lines analyzed and the generation upon which experiments were performed. For gene-edited lines, describe the editor used, the endogenous sequence targeted for editing, the targeting guide RNA sequence (if applicable) and how the editor was applied.*
Authentication	*Describe any authentication procedures for each seed stock used or novel genotype generated. Describe any experiments used to assess the effect of a mutation and, where applicable, how potential secondary effects (e.g. second site T-DNA insertions, mosiacism, off-target gene editing) were examined.*

Flow Cytometry

Plots

Confirm that:

☒ The axis labels state the marker and fluorochrome used (e.g. CD4-FITC).

☒ The axis scales are clearly visible. Include numbers along axes only for bottom left plot of group (a 'group' is an analysis of identical markers).

☒ All plots are contour plots with outliers or pseudocolor plots.

☒ A numerical value for number of cells or percentage (with statistics) is provided.

Methodology

Sample preparation	The tissue samples were passed through 200-mesh nylon mesh filters to obtain single-cell suspensions.<br><br>For all samples, cells were first stained with antibodies against surface antigens. In some experiments, cells were subsequently fixed, permeablized, and stained for intracellular antigens.<br><br>The detailed sample preparation method could be found in the article file and the supplementary information.
Instrument	NovoCyte 2070R, ACEA Biosciences Inc., USA
Software	NovoExpress (version 1.5.0), FlowJo (version 10)
Cell population abundance	No sorting was performed.
Gating strategy	Generally, cells were first gated on FSC/SSC. Singlet cells were gated using SSC-H and SSC-A. Dead cells were excluded and surface and intracellular antigen gating was performed on the singlet cell population. The cell populations were then

analyzed based on the expression of markers. Gating was then based on positive level. The detailed gating strategy could be found in the supplementary information.

☒ Tick this box to confirm that a figure exemplifying the gating strategy is provided in the Supplementary Information.
